# Transcatheter aortic valve replacement in patients undergoing robotic totally endoscopic coronary artery bypass: A case series

**DOI:** 10.3389/fcvm.2022.988029

**Published:** 2022-09-12

**Authors:** Ankur Srivastava, Jennifer Smazil, Lauren Roark, Hayla A. Shah, Husam H. Balkhy, Atman P. Shah

**Affiliations:** ^1^Section of Cardiology, University of Chicago Medicine, Chicago, IL, United States; ^2^University of Chicago Laboratory Schools, Chicago, IL, United States; ^3^Section of Cardiac Surgery, University of Chicago Medicine, Chicago, IL, United States

**Keywords:** TAVR, TECAB, CAD, survival, SAVR

## Abstract

Transcatheter aortic valve replacement (TAVR) has been utilized to treat patients with symptomatic aortic stenosis (AS). Recent trials suggest comparable efficacy compared to surgical aortic valve replacement (SAVR). Robotic off-pump totally endoscopic coronary artery bypass graft surgery (TECAB) had been shown to be a minimally invasive revascularization strategy with clinical results comparable to traditional coronary artery bypass graft surgery (CABG). Traditionally, pre-surgical coronary evaluation is considered necessary to optimize coronary revascularization at the time of AVR. The 2020 ACC/AHA Guideline for the Management of Patients with Valvular Disease gives a moderate recommendation, based on limited data, for CABG at the time of AVR in patients with significant coronary artery disease (CAD). This paper presents two patients with known significant CAD awaiting robotic TECAB who were treated with TAVR, prior to surgical revascularization. Robotic TECAB is unique in that it offers patients the ability to have complete coronary revascularization without a sternotomy and with early ambulation, discharge, and recovery. The case series demonstrates a hybrid approach that offers complete sternotomy sparing cardiovascular care to treat severe symptomatic AS and CAD. Since patients with severe aortic stenosis are at high risk of developing cardiac arrest and cardiogenic shock upon induction of anesthesia, the ability to treat severe symptomatic AS with TAVR under conscious sedation prior to TECAB can be considered as a safe an effective treatment.

## Introduction

### Patient 1

Patient 1 is a 74-year-old male with severe AS and significant CAD. He was self-referred for a sternal sparing approach to his combined disease after being offered sternotomy at another hospital. Transesophageal echocardiography (TEE) showed an ejection fraction (EF) of 55%, aortic valve area (AVA) of 0.6 cm^2^ and a mean gradient of 43 mmHg. Left heart catheterization (LHC) revealed a 70% stenosis in the proximal left anterior descending artery (LAD) with a 90% stenosis of the first OM and a totally occluded second OM with good targets for surgical revascularization. After a multidisciplinary meeting, the decision was made to complete TAVR before TECAB. Pre-TAVR, the patient had dyspnea and fatigue after one flight of stairs (NYHA class II) with an STS Risk Score of 10.708%.

The patient underwent successful TAVR with an Edwards Sapien S3 transcatheter valve via the right common femoral artery (Edwards Life Sciences, Irvine CA). Echocardiogram revealed a transvalvular mean gradient of 1 mmHg and no paravalvular leak. The patient had an uncomplicated hospital course post-TAVR and was discharged home the next day. At 1-month follow-up, he reported improvement of symptoms (NYHA class I). TTE revealed an EF of 64.2% with a mean gradient of 7 mmHg, without AI.

**Figure 1:**
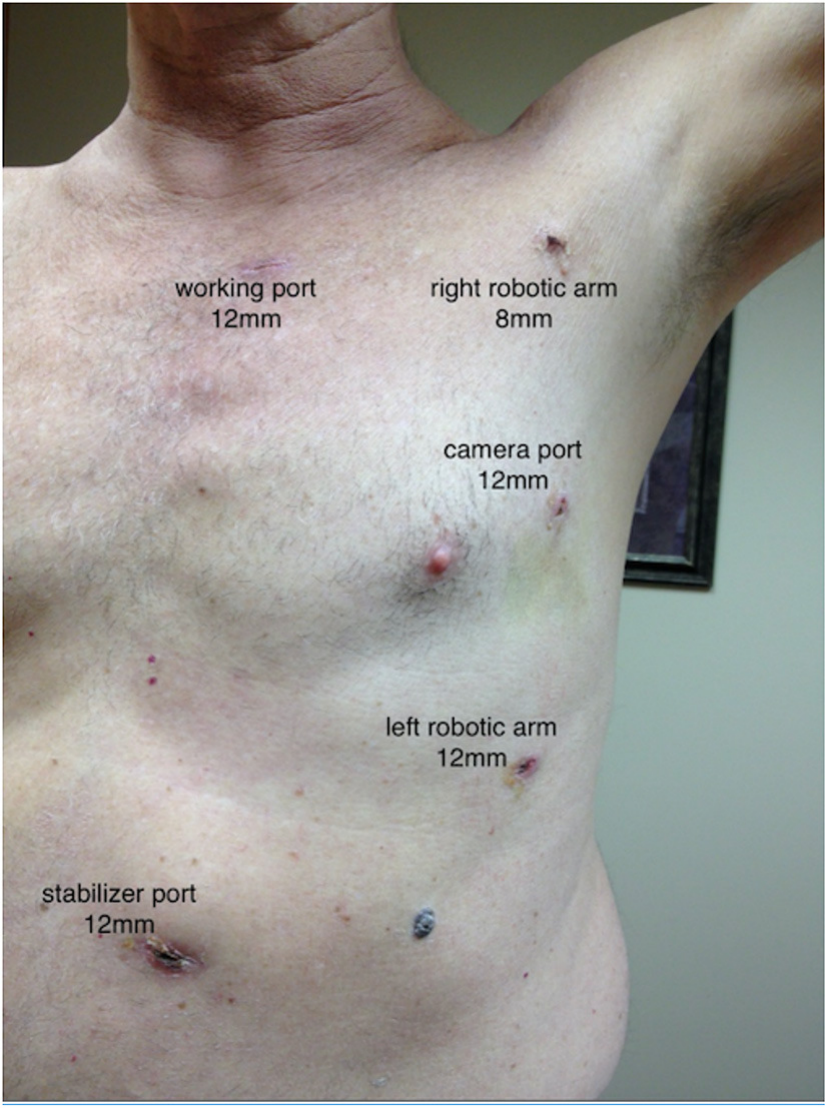
Illustration of TECAB approach (courtesy of Dr. H. Balkhy).

Two months later, the patient underwent a robotic 3-vessel TECAB after his DAPT was discontinued, and he received a RIMA-LAD (due to larger caliber vessel) with a sequential LIMA-OM1-OM2. Robotic TECAB was performed totally endoscopically (Figure 1). The DaVinci Robotic Surgical System (Intuitive Surgical, Sunnyvale CA) is used to endoscopically (no thoracotomy) harvest one or both internal thoracic arteries (ITA) as well as to perform the anastomosis is which is completed in our program off cardiopulmonary bypass on the beating heart.

The patient was discharged on post-op day two and reported resumption of all normal activities at post-op clinic visit with NYHA Class I symptoms.

### Patient 2

Patient 2 is a 67-year-old male with CAD who presented as a self-referral for severe AS/CAD. TTE showed an EF> 70%, AVA 1.2 cm^2^ with a mean gradient of 47.7 mmHg. Coronary angiography revealed a 70% proximal LAD that involved the ostium of the first diagonal branch and a 75% stenosis in the right coronary artery. Pre-TAVR, the patient reported fatigue with dyspnea on exertion while performing activities of daily living. His exercise tolerance was limited to 3 blocks, putting him in NYHA Class II with an STS risk of 1.9%. Given his obesity (BMI 42), a sternotomy was felt to be too high risk and he was referred for a sternal sparing approach.

The patient was treated with percutaneous coronary intervention of the RCA with a drug eluting stent to treat the 75% RCA lesion and then underwent uncomplicated TAVR with a 26 mm Sapien S3 transcatheter valve via a transfemoral approach. He had an uncomplicated hospital course and was discharged the following day. The patient returned to clinic in 1 month and reported slight improvement in shortness of breath. His follow up TTE revealed an of EF 68.7% with a trans-prosthetic gradient of 16.7 mmHg and mild peri-prosthetic regurgitation.

Robotic 2-vessel TECAB was completed 2 months later with LIMA-D1-LAD. His post-operative course was unremarkable and he reported resumption of pre-procedure activities with minimal complaints.

## Discussion

The 2020 ACC/AHA guidelines have deemed it reasonable to consider PCI before TAVR in patients with significant left main disease. However, in patients with multivessel disease, SAVR and CABG are recommended over TAVI and PCI (1). This is grounded in a theoretical concern that coronary ischemia will complicate aortic valve surgery with the thought that severe AS along with severe CAD would lead to hemodynamic compromise during induction of anesthesia and would result in a more complicated peri-operative course. Similarly, the European Society of Cardiology and European Association for Cardio-Thoracic Surgery recommend coronary angiography before valve surgery in patients with severe aortic stenosis and a history or concern for myocardial ischemia (2). However, the definition of significant CAD is dependent on angiographic assessment rather than functional assessment.

There is a need for more studies to support TAVR being completed before revascularization. In a meta-analysis of 15 studies, CAD was associated with higher all-cause mortality at 1 year in patients undergoing TAVR (3). Studies have found that patients who underwent incomplete revascularization as part of the TAVR workup had a higher incidence of cardiovascular events at 2 years, suggesting the importance of a revascularization strategy around the time of aortic valve surgery, (4). In patients undergoing SAVR, with co-existing CAD, Thalji demonstrated a decreased mortality after undergoing concomitant CABG at 5 and 8 years compared to those whose CAD was managed medically (5). After a multidisciplinary discussion, we decided to proceed with the TAVR prior to the TECAB because of the risk of hemodynamic collapse in patients with severe AS at the time of anesthesia induction.

While no randomized trial currently exists studying the role of surgical revascularization prior to SAVR, the ACTIVATION trial reported no benefit to PCI prior to TAVR when looking at 1 year mortality or rehospitalization (6). It also found increased bleeding in patients who underwent PCI (6). Given the purported benefit of fractional flow reserve (FFR) calculations prior to CABG, the ongoing NOTION-3 trial, which is investigating the role of FFR guided revascularization pre-TAVR, may provide additional insights on the timing of revascularization in the setting of AVR (7–9).

Currently, no large randomized trials comparing TECAB to traditional CABG currently exists, but there are a number of non-randomized trials and observational studies that suggest that TECAB is as safe and effective as traditional CABG (10, 11). And further studies are needed to assess the cost-effectiveness of this strategy. The up-front costs of the TAVR and TECAB may be higher than traditional surgery, however, the shortened hospital stay and potentially fewer complications may offset that initial cost.

One additional concern is the duration of antiplatelet therapy after TAVR. Currently, guidelines suggest 6 months of dual antiplatelet therapy (at least aspirin 81 milligrams daily and clopidogrel 75 milligrams daily) after TAVR. However, a number of studies have suggested a shorter duration is safe and effective (8). Therefore, we elected to treat patients with 1 month of dual antiplatelet therapy in advance of their surgical revascularization.

Our hospital is a referral center for robotic heart surgery and routinely offers single and multivessel totally endoscopic all arterial revascularization for appropriate candidates. As seen in Figure 1, our approach for patients with CAD combined with AS demonstrates that TAVR can be safely completed prior to coronary revascularization (12, 13). They returned to their pre-procedural activities within a few weeks of each procedure respectively and felt improvement of their previous symptoms. These patients will have continuous clinical follow-up to fully assess the clinical efficacy and safety of this combined strategy.

## Conclusion

This case series reports that TAVR prior to robotic TECAB offers a safe sternal-sparing approach to patients with combined aortic valve and coronary artery disease who are unwilling or unable to undergo a standard sternotomy (Data in Tables 1, 2). This hybrid technique offers a truly minimally invasive approach to patients with AS and CAD that is not amenable to percutaneous revascularization. Early discharge and recovery are hallmarks of this approach and further studies are needed to not only elucidate the role of coronary revascularization prior to TAVR/SAVR, but also to investigate the hybrid role of TAVR and TECAB.

**Table 1:** Clinical characteristics.

	**Age**	**Comorbidities**	**Laboratory data**	**Smoker**	**Prior revascularization**
Patient 1 (BB)	74	Diabetes	Creatinine 0.9 mg/DL	Yes	None
		Hypertension	Hb (g/dL)/HCT (%): 11.2/39		
Patient 2 (WF)	67	Diabetes	Creatinine 1.3 mg/DL	Yes	None
		Hypertension	Hb (g/dL)/HCT (%): 10.6/35		
		Dyslipidemia			

Summary of cases by angiography results (*LAD* left anterior descending; *OM* obtuse marginal; *D1* 1st diagonal; *RCA* right coronary), valve hemodynamics, left ventricular ejection fraction, valve type, TECAB results and 90-day survival.

**Table 2:** Summary of cases.

	**Age**	**Angiography**	**Aortic valve**	**LVEF (%)**	**Valve type**	**TECAB**	**90 Day survival**
Patient 1 (BB)	74	LAD 70%	Mean gradient: 41.9 mmHg	73	Edwards Sapien S3 Ultra 26 mm	RIMA—LAD	Yes
		OM1 90%	Velocity: 3.6 M/sec			Sequential LIMA-OM1/OM2	
		OM2 100%	AVA: 0.76 cm^2^				
Patient 2 (WF)	67	LAD 70%	Mean Gradient: 47.4 mmHg	>70	Edwards Sapien S3 Ultra 26 mm	LIMA—LAD/D1	Yes
		D1 80%	Velocity: 4.6 M/sec				
		RCA 75%	AVA: 1.2 cm^2^				

## Data availability statement

The original contributions presented in the study are included in the article/supplementary material, further inquiries can be directed to the corresponding author/s.

## Ethics statement

Written informed consent was obtained from the individual(s) for the publication of any potentially identifiable images or data included in this article.

## Author contributions

ASh, ASr, and HB were responsible for the design of this study, data collection, manuscript creation, and manuscript review. JS, LR, and HS were responsible for manuscript creation and review. All authors contributed to the article and approved the submitted version.

## Conflict of interest

The authors declare that the research was conducted in the absence of any commercial or financial relationships that could be construed as a potential conflict of interest.

## Publisher's note

All claims expressed in this article are solely those of the authors and do not necessarily represent those of their affiliated organizations, or those of the publisher, the editors and the reviewers. Any product that may be evaluated in this article, or claim that may be made by its manufacturer, is not guaranteed or endorsed by the publisher.
